# Pain and Empathy: The Effect of Self-Oriented Feelings on the Detection of Painful Facial Expressions

**DOI:** 10.1371/journal.pone.0100434

**Published:** 2014-07-01

**Authors:** Delphine Grynberg, Pierre Maurage

**Affiliations:** 1 Psychological Sciences Research Institute, Université Catholique de Louvain, Louvain-la-Neuve, Belgium; 2 Laboratory for Experimental Psychopathology, Psychological Sciences Research Institute, Université Catholique de Louvain, Louvain-la-Neuve, Belgium; University of Bologna, Italy

## Abstract

**Background:**

Painful facial expressions have been shown to trigger affective responses among observers. However, there is so far no clear indication about the self- or other-oriented nature of these feelings. The purpose of this study was to assess whether facial expressions of pain are unconsciously associated with other-oriented feelings (empathic concern) or with self-oriented feelings (personal distress).

**Method:**

70 participants took part in a priming paradigm in which ambiguous facial expressions of pain were primed by words related to empathic concern, distress, negative or by neutral words. It was hypothesized that empathic concern or distress-related words might facilitate the detection of pain in ambiguous facial expressions of pain, independently of a mere effect of prime (i.e., neutral words) or an effect of valence congruency (negative primes).

**Results:**

The results showed an effect of prime on the detection and on the reaction time to answer “pain” when confronted to ambiguous facial expressions of pain. More specifically, the detection of pain was higher and faster when preceded by distress primes relative to either neutral or negative primes.

**Conclusion:**

The present study suggests that painful expressions are unconsciously related to self-oriented feelings of distress and that their threat value might account for this effect. These findings thus shed new light on the automatic relationship between painful expressions and the affective components of empathy.

## Introduction

Pain is defined as an unpleasant sensory and emotional experience associated with actual or potential body damage. Painful feelings can be expressed by various ways (e.g., facial expressions, voice prosody), and it has been suggested that witnessing someone expressing pain triggers empathic affective responses in the observer (e.g., [1]). Nevertheless, the exact nature of these affective responses remains largely undetermined, notably concerning their orientation. Particularly, it is still unclear whether these responses to pain are mostly oriented towards the self or towards the others. Self-oriented responses can be globally defined as feelings of discomfort and distress focusing on the reduction of the observer's own distress when witnessing another's negative experience, while other-oriented responses are warmth and empathic concern focusing on the other's well- being [2]. So far, studies have mainly investigated the role of moderators on state empathic affective responses (distress versus empathic concern) for someone in pain (e.g., [3]) or the association between trait measures of empathic affective responses and neural activation in response to painful facial expressions [4], [5]. In terms of situational empathic affective responses, only one study has investigated the influence of pain appraisal and perspective taking on situational affective empathic responses to painful expression [1]. This study has shown that (1) imagining oneself triggers distress while imagining another person triggers empathic concern; (2) when imagining the other person, empathic concern responses are positively correlated with the anterior medial cingulate cortex activation (involved in the affective dimension of pain). However, there are no empirical evidence that painful expressions are automatically associated with distress or empathic concern in general. This question is relevant mainly because distress and empathic concern involve different behavioural consequences: distress is *oriented to the self* and might motivate individuals to *avoid* the source of the threat and thus the person itself while empathic concern is *oriented to others* and might make one more available to care for others and thus to *approach* this person [6].

Related to automatic avoiding or approaching pain facial expressions, Yamada and Decety [7] investigated if the detection of pain in ambiguous expressions of pain (50% pain and 50% happiness) was facilitated when primed by dislikeable words (e.g., liar, hypothesized to be associated with avoidance) or likable words (e.g., honest, hypothesized to be associated with approach) compared to scrambled words or to no prime at all. Using signal detection theory, they showed that the criterion to judge ambiguous faces as expressing pain was significantly different from zero when the faces were primed by dislikeable words. This suggests that participants more often judged ambiguous expressions of pain and happiness as expressing pain when these expressions were primed by dislikeable words.

According to the authors, this supports that pain is automatically associated with avoidance from the threat value of pain and not with a motivation to approach toward the other in pain.

Therefore, if painful expressions activate the threat system, these expressions might also be associated with distress feelings rather than compassion feelings (see [8]). However, several methodological and statistical limits were related to this seminal study [8], [9]. Indeed, the main limitations of this paper were that: (1) the primes are not directly associated with avoidance/approach or affective empathic responses, (2) the design does not allow to refute any facilitation effect resulting from a valence congruency, and (3) the calculation of the criterion score as a detection signal index while this latter is independent of the effect of the priming (see the Method section for a more detailed explanation). Therefore, the findings are difficult to interpret in terms of avoidance or approach motivation and respectively in terms of distress or empathic concern.

In order to understand how painful expressions are automatically associated with empathic affective responses, we will address these limitations (1) by presenting distress and empathic concern primes, (2) by controlling for other variables (i.e., negative and neutral primes *and* targets), and (3) by using only the detection signal index that is relevant with this paradigm (i.e., sensitivity scores). More specifically, the present study will explore three unresolved questions: Are ambiguous painful expressions more associated with self-oriented or other-oriented feelings (*Aim 1*)? If ambiguous painful expressions are associated with these feelings, is it due to an effect of prime (*Aim 2*) and/or to an effect of valence congruency (*Aim 3*)?

In sum, this study aims to investigate three different but related effects on the detection of pain in ambiguous expressions of pain (in terms of responses and reaction times): The effect of self-oriented versus other-oriented feelings on the detection of pain in ambiguous expressions of pain (distress versus empathic concern primes) (*Aim 1*) by controlling for the effect of prime (distress versus neutral primes; empathic concern versus neutral primes) (*Aim 2*) and for the effect of valence congruency (distress versus negative primes) (*Aim 3*).

## Method

### Participants

Seventy students in Psychology at the Université Catholique de Louvain (52 females) took part in the study. They were aged from 18 to 31 (*M* = 21.70; *SD* = 2.00). They were paid 8 Euros for their participation. We obtained written informed consent from each participant, which was approved by the ethical committee of the Psychological Sciences Research Institute (Université Catholique de Louvain).

### Material

#### Pre-test

**Figure 1:**
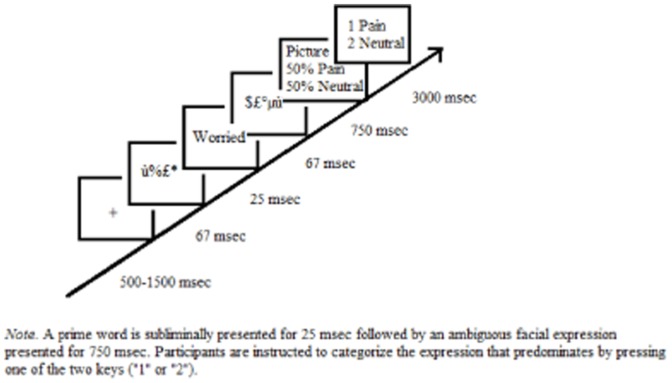
Schematic overview of a typical trial.

A pretest phase was conducted in order to select 24 targets and 48 fillers of ambiguous facial expressions. For these pre-tests, the paradigm was based on Yamada and Decety's [7] study (see Fig. 1). In the original paradigm, participants were asked to complete the priming task in which prime words were subliminally presented for 25 msec. Each trial started with a fixation cross (presented for a duration that varied between 1000 and 3000 msec). This was followed by (1) hash-mask symbols (67 msec), (2) the prime (25 msec), (3) ampersands backward mask (67 msec), and (4) by an ambiguous facial expression of pain morphed with happiness (750 msec). Participants had then 3000 msec to categorize the face as pain or no-pain. In these pre-tests, we used the same paradigm except that symbols were used for mask *and* prime stimuli. Furthermore, additionally to ambiguous facial expression of pain morphed with happiness, we also presented ambiguous expressions of pain morphed with neutral and fearful expressions, ambiguous expressions of fear morphed with neutral and happy expressions, and ambiguous expressions of happiness morphed with neutral expressions. These morphed expressions were based on 16 original pictures from 4 different actors expressing 4 emotions (fear, pain, happy, or neutral) [10]. The pictures came from the videos of Simon et al.'s [10] validated battery. For each emotion and for each actor, we have selected the frame that was the most expressive and transformed it into a picture. All the pictures were grayscaled. The faces were then morphed with the program Morphman 2000 in order to obtain six expressions continua (fear-neutral, happiness-neutral, happiness-fear, pain-neutral, pain-happiness and pain-fear).

In the pretest phase, 99 volunteers (76 females) were informed that pictures of emotional and neutral facial expressions will be presented and were instructed to categorize the expression that predominates by pressing one of the two keys (“1” or “2”). The instructions were as follow: For each trial, you will see a face. Your task will be to assess which emotion predominates. By pressing “1” or “2”, you will have 3 seconds to decide which emotional expression predominates.

Because of the difficulty to find targets and fillers that correspond to our criteria (cf Stimuli section), four different sessions were necessary. The successive sessions only included the expressions that were missing from the set of stimuli used in the actual experiment (i.e., 24 targets and 48 fillers). Therefore, the four different sessions were composed of different sets of pictures. Furthermore, the sample of participants differed within each session. Session 1 included 20 participants (15 females) (*M_Age_* = 22.00; *SD_Age_* = 1.89), Session 2 included 60 participants (51 females) (*M_Age_* = 20.65; *SD_Age_* = 2.25), Session 3 included 11 participants (6 females) (*M_Age_* = 24.72; *SD_Age_* = 3.00), and Session 4 included 8 participants (4 females) (*M_Age_* = 27.37; *SD_Age_* = 4.59).

#### Stimuli

The ambiguous targets were chosen if 50% of the participants (from the pre-test phase) detected one expression and if the other 50% of participants detected the other expression. Because we have six expressions continua and four different actors, there are 24 ambiguous targets. The fillers were also morphed faces, but with a rate detection of 60% -40% (24 ambiguous fillers) and 40%–60% (24 ambiguous fillers). The fillers were only used in order to reduce the salience of critical stimuli (i.e., targets), and will therefore not be analyzed.

#### Primes

The primed words were selected from a database that collected norms for words involving subjective (valence, arousal, imageability and concreteness) and objective (length, lexical frequency and complexity) dimensions [11]. There were four neutral (e.g., salute), four negative (e.g., discouragement), four distress (e.g., worried) or four empathic concern (e.g., tender) words. The distress and empathic concern words were partly based upon the research of Batson et al. [2] while no word from the negative or neutral category was based on it. Nonparametric tests revealed that based on these norms, the four categories were similar in terms of length, lexical frequency, complexity, imageability, and concreteness (*ps*>.08). The distress and negative words did not differ in terms of valence (*p* = .90), but were more negative than neutral words and empathic concern words (*ps*<.001). Empathic concern words were more positive than neutral words (*p*<.001). In terms of arousal, neutral words were less arousing than empathic concern, negative words and distress words (*ps*<.001). These three latter categories did not differ from each other for arousal (*p*>.25).

### Procedure

The instructions and the design of the main experiment were identical to the pre-test except that the primes were words and not symbols (see Fig. 1). Participants were requested to perform the priming task, in which prime words were subliminally presented for 25 msec followed by an ambiguous facial expression presented for 750 msec. Participants were instructed to categorize the expressed emotion. There were 6 blocks (one for each type of morphing) of 96 trials each [for the target 50%–50%: 16 trials per category of prime (16*4 trials); for the fillers 40%–60%: 4 trials per category of prime (4*4 trials); for the fillers 60%-40%: 4 trials per category of prime (4*4 trials)]. Because our main focus was the 50%–50% stimuli and because the experiment lasted around 35 minutes, we wanted to reduce as much as possible the number of less relevant stimuli (40%–60% and 60%-40%). Therefore, we decided to present less fillers than targets.

### Statistical analyses

Signal Detection analysis (see [7]) was used in order to investigate the sensitivity of pain detection in 50%–50% ambiguous painful facial expressions. However, contrary to Yamada and Decety's [7] methodology, we have decided to avoid presenting a block without primes in order to keep participants' attentional focus intact. Furthermore, we have chosen to measure the sensitivity (d-prime) only and not criterion (C) because this latter reflects a response bias that is independent of the priming effect (see [9]). Yamada and Decety [7] indeed used two indices of detection signal theory: the sensitivity and criterion indices. The *sensitivity* score corresponded to the difference between hits (“pain” response when faces are preceded by primes) and false alarm (“pain” response when faces are not preceded by primes) and the *criterion* corresponded to the sum of these false alarm and hits. The criterion thus refers to trials that are primed (hits) and unprimed (false alarms). Therefore, in the present study, (1) a “hit” corresponded to a “pain” response to ambiguous facial expressions of pain morphed with happiness, fear or neutral when primed with distress words; (2) a “miss” corresponded to a “no-pain” response to the same trials; (3) a “false alarm” corresponded to a “pain” response to facial expressions of pain morphed with happiness, fear or neutral when primed with compassion, negative or neutral words; (4) a “correct rejection” corresponded to a “no-pain” response to the same trials. The fear-neutral, happy-neutral, and fear-happy blocks were added only to reduce the salience of critical stimuli (i.e., targets) and will therefore not be analyzed.

For each block (i.e., pain-neutral, pain-fear, pain-happiness), a sensitivity score [d-prime = Z(hit)- Z(false alarm)] was calculated to measure the sensitivity to the presence of pain when primed with distress words. If the sensitivity score that refers to the sensitivity to detect pain in ambiguous facial expression of pain after distress words (hits) relative to (1) empathic concern words [Z(Hit_Distress) – Z(False Alarms_Empathic_Concern)], (2) neutral words [Z(Hit_Distress) – Z(False Alarms_Neutral)], or (3) negative words [Z(Hit_Distress) – Z(False Alarms_Negative)], is positive, this will suggest that painful expressions are associated with self-oriented feelings (1) to a greater extent than other-oriented feelings (2) independently of a priming effect or (3) independently of a valence congruency effect. In terms of reaction times, a similar conclusion can be drawn if the difference between the reaction times to detect pain in ambiguous painful expressions after self-oriented feelings and reaction times to detect pain in painful expressions after empathic concern words, neutral words or negative words is significantly different from zero: positive values will suggest that painful expressions are associated with self-oriented feelings (1) to a greater extent than other-oriented feelings (2) independently of a priming effect or (3) independently of a valence congruency effect.

In order to investigate whether compassion words facilitate the detection of pain, a sensitivity score was also calculated for compassion primes: a “hit” corresponded to a “pain” response to ambiguous facial expressions of pain morphed with happiness, fear or neutral when primed with compassion words; a “false alarm” corresponded to a “pain” response in to facial expressions of pain morphed with happiness, fear or neutral when primed with neutral words) [Z(Hit_Empathic_Concern) – Z(False Alarms_Neutral)]. Regarding the analysis of reaction times (RTs) in responses to 50%–50% ambiguous painful facial expressions, we have subtracted the RTs to detect pain after compassion, negative or neutral primes from the RTs to detect pain after distress primes. We have also subtracted the RTs to detect pain after neutral primes from the RTs to detect pain after compassion primes.

Similarly to Yamada and Decety [7], the effects were compared to zero by using one-sample t tests, with a significance level at *p*<.05. The data are deposited in a publicly available database (http://essenselab.wordpress.com/material/).

## Results

### Responses

The average detection scores for each pain block (and for each prime) are presented in Table 1. The signal detection analysis revealed that relative to *neutral words*, the pain sensitivity score to ambiguous painful faces primed with *distress words* was significantly above-chance level in the block fear-pain (*M _sensitivity_* = 0.10, *SD_sensitivity_* = 0.39) (*t*(66) = 2.03 *p* = .046; Cohen's d = .50; Table 2). In other words, when ambiguous painful expressions are morphed with fear, participants detect more often pain when these expressions are primed with distress words relative to neutral words. The other sensitivity scores for ambiguous painful expressions were not significantly above-chance level (*ps*>.054).The sensitivity scores for the other ambiguous expressions (fear-happy; fear-neutral; happy-neutral) were not significantly above-chance level (*ps*>.28).

**Table 1:** Descriptive data (Mean and Standard Deviation) of “pain” responses and reaction times to ambiguous expressions of pain (50%–50%) for each type of prime.

		Continuum											
		Pain - Happiness				Pain - Neutral				Pain – Fear			
		Response		RTs (ms)		Response		RTs (ms)		Response		RTs (ms)	
		M	SD	M	SD	M	SD	M	SD	M	SD	M	SD
Prime	Empathic Concern	8.51	3.53	1306.04	217.75	10.13	3.01	1185.60	260.20	5.46	3.37	1294.00	298.91
	Distress	8.49	3.31	1273.24	213.80	9.67	3.27	1149.23	167.60	5.70	3.35	1346.78	322.45
	Neutral	8.69	3.36	1271.27	209.97	10.04	3.31	1181.70	230.58	5.13	2.97	1329.52	280.69
	Negative	8.58	3.42	1246.85	236.24	9.97	2.98	1213.80	231.78	5.48	3.28	1308.85	250.69
	Mean	8.57	3.41	1272.67	176.73	9.96	3.14	1182.58	197.44	5.44	3.24	1311.64	232.33

*Note*. The total of responses for each category of prime is 16.

**Table 2:** One-sample t tests for each type of ambiguous expression of pain (i.e., morphed with happiness, neutral, and fearful expressions) and for each contrast.

Morphing	Contrast (Primes)	Responses	Reaction times
		*t* (66)	t (65)
Pain- Happiness	Distress - Compassion	−0.15	−1.87
	Distress - Neutral	−0.15	−0.23
	Distress - Negative	−0.50	0.94
Pain- Neutral	Distress - Compassion	−1.96	−1.54
	Distress - Neutral	−1.61	−1.69
	Distress - Negative	−1.21	−2.95***
Pain- Fear	Distress - Compassion	0.94	1.29
	Distress - Neutral	2.03*	−0.07
	Distress - Negative	0.68	1.08

*Note*. For Responses, positive values correspond to higher sensitivity to detect pain in ambiguous expressions of pain primed with distress words relative to compassion, neutral or negative primes. For Reaction Times, negative values correspond to quicker detection of pain in ambiguous expressions of pain primed with distress words relative to compassion, neutral or negative primes.

* *p*<.05;

*** *p*<.005.

These results suggest that painful expressions are associated with self-oriented feelings independently of a mere effect of priming (Aim 2). However, painful expressions were not more particularly associated with self-oriented or with other-oriented feelings (Aim 1).

### Reaction times

The average detection RTs scores for each pain block (and for each prime) are presented in Table 1. The one-sample t test analysis revealed that the difference between the RTs to detect pain in ambiguous expressions of pain morphed with neutral expression when primed with distress words relative to negative words was different from zero (*M_difference_* = −64.57; *SD_difference_* = −178.93; *t*(66) = −.1.69; *p* = .004; Cohen's d = .42; Table 2). This suggests that participants are faster to detect pain in ambiguous expressions of pain morphed with neutral expression when these expressions are primed by distress words relative to negative words. The other difference scores were not significant (*ps*>.07). The difference scores for the other ambiguous expressions (fear-happy; fear-neutral; happy-neutral) were not significantly different from zero (*ps*>.05). These results suggest that painful expressions are associated with self-oriented feelings independently of an effect of valence congruency (Aim 3). However, these expressions are not more associated with self-oriented than with other-oriented feelings (Aim 1).

## Discussion

The main aim of the study was to test whether painful expressions are automatically associated with distress self-oriented feelings or rather with other-oriented feelings of compassion. So far, the topic has been mainly investigated at a neural level. This study is the first to investigate the question at a behavioural level in general (i.e., without focusing on possible moderators) with more adequate methodology and statistical analyses than Yamada and Decety's [7] study (1) by presenting distress and empathic concern primes, (2) by refuting any facilitation effect resulting from a valence congruency (i.e., presentation of negative primes and of ambiguous facial expressions of fear) and (3) by using the only detection signal index that is relevant with this paradigm (i.e., sensitivity scores).

This study fills thus the gap regarding the association between affective empathic responses and painful expressions. We used a priming task in which ambiguous facial expressions of pain were primed by distress and empathic concern words. More specifically, this study had three aims: (1) investigating if painful expressions are more associated with self-oriented or with other-oriented feelings (Aim 1); (2) investigating that the possible association between painful expressions with self-oriented feelings (or other-oriented feelings) is not due to the presence of primes (Aim 2); (3) investigating if painful expressions are associated with self-oriented feelings independently of an effect of valence congruency (Aim 3).

In order to refute any facilitation effect resulting from a valence congruency we have added negative and neutral primes, and other ambiguous facial expressions of arousing negative emotions (i.e., fear).

The findings revealed that relative to negative words primes, distress words primes facilitate the detection of pain in 50% pain-50% neutral expressions in terms of RTs (small effect size). We have also shown that relative to neutral primes, distress primes lead to greater sensitivity of pain in 50% pain-50% fear expressions (medium effect size). It is worth mentioning that no effect of prime was found for the other ambiguous expressions that did not include painful expressions (i.e., fear-neutral, fear-happy; happy-neutral). Our results thus showed that unconscious processing of distress words facilitates the detection of pain expression only relative to neutral and negative primes (Aim 2 and 3). The study of Yamada and Decety [7] has leaded to the same conclusion, by showing that the criterion to judge the ambiguous face as expressing pain was significantly different from zero when the faces where primed by dislikeable words. However, as suggested by Pfaller et al. [9], this index reflects a response bias that is independent of the priming effec). Therefore, the present study is the first to show that self-oriented feelings facilitate the detection of painful expressions. Regarding the underlying mechanisms, we argue that the facilitation effect of distress primes on the detection of pain in ambiguous facial expressions of pain might be accounted for by the threat-related value of painful expressions. The present study might provide evidence of an activation of a threat-detection mechanism when confronted with distress words. It has been suggested that painful expressions are associated with threat [12] and that threatening stimuli leads to distress feelings [13]. The hypothesis that painful facial expressions are associated to threat has been supported by recent findings showing that these expressions require attentional resources at very early time [12], which has been suggested to result from the activation of the threat detection system. Thus, based on these findings, we hypothesize that in the present study, the threat value of painful expressions might have been activated by the distress primes, accounting for the association between painful expressions target and distress primes.

Importantly, we did not show that self-oriented primes facilitate the processing of pain to a greater extent than other-oriented primes (i.e., compassion words; Aim 1). It can thus not be concluded that painful expressions are more associated with self-oriented relative to other oriented responses. The temporal course of these feelings might partly account for this result. We rather suggest that our findings might support the hypothesis that both self and other-oriented responses to someone expressing pain may occur but at different moments (see [8]). Distress responses might be automatically triggered, and might secondly turn into more regulated feelings of concern. Because of the more automatic nature of distress responses, it is presumed that these are not influenced by moderator factors. However, we assume that the association between painful expressions and empathic concern is less automatic and might thus be influenced by moderators (e.g., emotion regulation abilities). It is worth mentioning that because of the more complex nature of empathic concern, it may be impossible to unconsciously prime with empathic concern as this emotion might require longer time to process, and conscious processing.

Furthermore, the hypothesis that both responses might occur is in line with the fact that the exact nature of these responses to painful expressions (i.e., self or other oriented) is still unclear. In terms of neural activation in response to facial expressions of pain and its association with situational and dispositional measures of empathy, the results are inconsistent [14]. For instance, while Lamm et al.'s [1] study has shown that when participants have to imagine the other, there is a positive correlation between situational empathic concern responses and the activation in the anterior medial cingulate cortex (involved in the affective dimension of pain). Another study showed that the activation of the left anterior insula, also involved in the affective component of pain processing, correlated positively with dispositional measures of empathic concern but also of personal distress [5].

All together, these studies support that the processing of painful expressions is associated to affective empathic responses. However, future studies are needed to focus on the degree of automaticity of self and other-oriented responses. Furthermore, because we did not specifically investigate how painful expressions lead to self or other-oriented feelings, no conclusions can be drawn about the emotions elicited by pain in others. Future studies should thus also focus on the exact nature of the situational affective responses to someone expressing pain, at both subjective and neural levels, and to assess the possible moderators and change over time of these feelings.

One methodological constraint has to be considered. The results did not reveal similar effects of prime on ambiguous expressions of pain in terms of RTs and responses.

This effect might be accounted for by the possible presence of a speed accuracy trade-off. The descriptive data indeed show that while the condition with shorter latencies (pain-neutral) is characterized by greater detection of pain, the condition with longer latencies (pain-fear) is characterized by lower detection of pain. Therefore, the association between painful expressions and distress might have emerged in different ways depending on the morphing.

A second limitation refers to a possible sex effect. It has indeed been shown that females report higher empathy responses (e.g., [15]). Future studies should thus include more male participants and investigate if sex influences the detection of painful expressions.

Finally, one could argue that the neutral words present a slightly positive valence. This is based on the fact that primes were selected from a battery that provides norms for words that present a social dimension [11]. In this battery, the social dimension is defined as a behaviour, a thought, or a feeling from a person to another person. Therefore, the words cannot be totally neutral. However, the valence of these words was less positive than empathic concern and less negative than distress and negative primes. Furthermore, the neutral primes were also less arousing than all other primes.

## Conclusions

This study investigated for the first time if the activation of the concept of distress and empathic concern would automatically facilitate the processing of painful expressions. The present study globally confirms, in a controlled design, the hypothesis that self-oriented feelings of distress are associated with greater and rapid recognition of ambiguous painful expressions and supports the hypothesis that painful expressions may activate the avoidance system. These results are thus a first step towards a further exploration of the subjective and physiological aspects of these feelings in response to someone expressing pain.
